# Long-term Disease-free Survival Following Combination Multi-visceral and Metastatic Resection with Neoadjuvant FOLFIRINOX for Pancreatic Adenocarcinoma: A Case Report

**DOI:** 10.7759/cureus.429

**Published:** 2015-12-23

**Authors:** Mikael Sodergren, Kirsty Brammer, David Cunningham, Satvinder Mudan

**Affiliations:** 1 Department of Surgery and Cancer, Imperial College London; 2 Department of Academic Surgery, The Royal Marsden NHS Foundation Trust; 3 Department of Gastrointestinal Oncology, The Royal Marsden NHS Foundation Trust

**Keywords:** pancreatic adenocarcinoma, folfirinox, multi-visceral, metastatic, pancreatectomy

## Abstract

We describe a case of metastatic pancreatic adenocarcinoma treated with neoadjuvant FOLFIRINOX chemotherapy and combined pancreatic multi-visceral and metastatic liver resection in a patient currently disease-free four years after diagnosis.

## Introduction

Metastatic pancreatic adenocarcinoma is associated with a poor prognosis. Recent advances in chemotherapy have led to some improvement in prognosis; however, reports of long-term survival are rare.

## Case presentation

**Figure 1:**
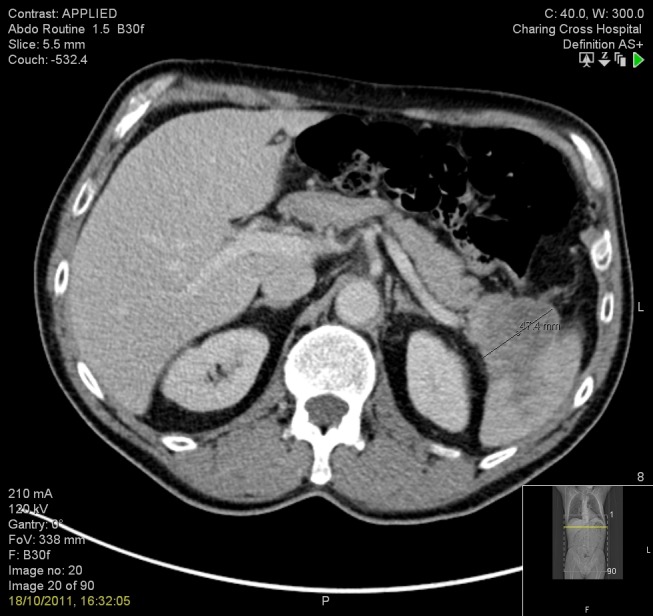
CT showing primary pancreatic tumour

We describe the case of a 63-year-old Professor of rheumatology who presented in October 2011 with a single episode of acute, left-sided back pain and microscopic haematuria. As part of these investigations, he underwent a non-contrast CT which showed no evidence of renal tract calculi. A subsequent contrast-enhanced CT demonstrated the presence of a 4.7 cm heterogeneous but predominantly low-attenuation mass arising from the tail of the pancreas and extending towards the splenic hilum. On the cross-sectional imaging there was loss of the fat plane between the mass and the spleen suggesting direct invasion as illustrated in Figure 1.

**Figure 2:**
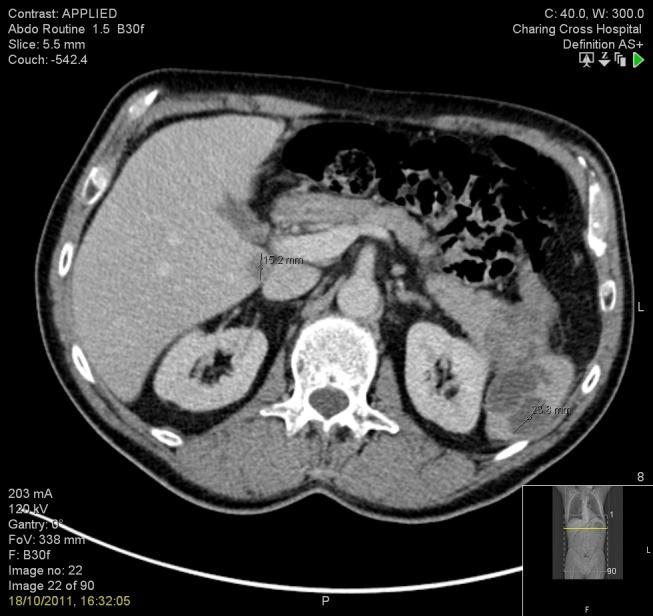
CT showing liver metastasis in segment VI

Furthermore, there was a 1.5 cm low-attenuation area within the medial aspect of segment VI of the liver likely representing a metastatic deposit, as seen in Figure 2.

Subsequent investigations included a PET scan, which demonstrated a fluorodeoxyglucose (FDG) positive pancreatic tail primary with at least one liver metastatic deposit in segment 6. He was discussed in the regional hepatobiliary multidisciplinary team (MDT) meeting, where at the end of October 2011, a CT-guided biopsy of the pancreatic tail mass was arranged. Histology confirmed an infiltrative tumor comprising malignant glands, composed of medium-sized cells with abundant cytoplasm but no cytoplasmic inclusions. There was an increased nuclear cytoplasmic ratio; the nuclei were pleomorphic with lobulated to smooth outlines but no nuclear grooves. The nuclear chromatin was evenly dispersed, and each nucleus had a single, prominent, peripherally located nucleolus. Numerous atypical mitotic figures were seen, and a mitotic count of 1-5 mitotic figures per high power field was noted. The features were consistent with a poorly differentiated pancreatic ductal adenocarcinoma. Following further discussion in the MDT, he was commenced on a 6 cycle course of FOLFIRINOX chemotherapy starting 23 November 2011. His CA 19-9 fell from 23 to less than 2 on completion of his chemotherapy. His post-chemotherapy imaging (CT and MRI) revealed a reduction in size of both the primary and the contiguous deposit related to the spleen and the liver metastasis of approximately 50%. It was decided he would receive another 3 cycles of FOLFIRONOX with a view to gaining further systemic response.

Following a total of nine cycles of chemotherapy, he was again discussed in the regional MDT, where it was noted that radiologically there had been a significant response; the liver lesion was no longer visible, and the splenic hilar disease was reduced. He had an incidental new pulmonary emboli in the right middle and bilateral lower lobe pulmonary arteries and was commenced on therapeutic, low-molecular-weight heparin--although symptomatically he suffered only grade 1 shortness of breath on exertion.

On 28 March 2012, he underwent an open distal pancreatectomy, a splenectomy, a transverse colectomy, and a liver resection of segment 5/6. His post-operative recovery was uncomplicated, and he was discharged on day 9.

Histology confirmed a poorly differentiated desmoplastic adenoma with some chemotherapeutic regression and areas of central inflammation due to associated pancreatitis and ductal rupture. The residual carcinoma invaded the capsular spleen into the parenchyma with focal angioinvasion. There was intense fibrosis surrounding the tumor which extended to the peritoneal surface, but there was no peritoneal breach by viable tumor. There was invasion of the colonic mesentery, and viable tumor was present in the muscularis propria, although the resection margins were free from tumor. One lymph node was involved by tumor, probably by direct invasion, but seven other lymph nodes were uninvolved. The mid-pancreas and resection margin of the pancreas were free from tumor. The hepatic parenchymal resection contained metastatic poorly differentiated desmoplastic adenocarcinoma abutting the capsule but not penetrating it, with the margins free from tumor. This was staged as pT3N1M1 V1 R0 poorly differentiated desmoplastic ductal adenocarcinoma of the pancreas with chemotherapeutic regression.

He underwent 3 cycles of adjuvant FOLFIRINOX, which was completed in June 2012, and he has remained well since his surgery with ongoing but improving grade 1 neuropathy affecting his fingertips and toes. He has undergone regular surveillance imaging, and his latest CT in October 2015, four years following diagnosis, revealed no evidence of disease recurrence or distant metastases. 

## Discussion

Metastatic pancreatic adenocarcinoma has a dismal prognosis with a median survival of around only 7 months when treated with standard, gemcitabine chemotherapy alone [1]. Traditionally, metastatic disease has been a contraindication to surgery due to poor outcomes; nonetheless, a recent systematic review of the evidence concerning liver-only metastases (consisting mainly of case series) has suggested that overall survival with one or few metastases that are concomitantly resected seems to be comparable to cases with no evidence of metastases [2]. This clearly needs to be considered in light of the quality of the evidence, and this approach has not gained widespread adoption. Furthermore, multi-visceral resection for pancreatic adenocarcinoma has remained controversial; however, there is data to indicate that this approach has similar overall survival but higher morbidity compared with standard pancreatic resection [3].

The landmark PRODIGE 4/ACCORD 11 trial [1] showed a significantly improved overall survival of patients with metastatic pancreatic adenocarcinoma receiving FOLFIRINOX chemotherapy compared with gemcitabine at the cost of higher toxicity. Nevertheless, there have been some excellent case results reported in the literature including those with a complete pathological response [4].

At the Royal Marsden, we have used FOLFIRINOX for the past 5 years for metastatic adenocarcinoma of the pancreas for patients with good performance status and also in the setting of neoadjuvant therapy for tumor downstaging in locally advanced disease [5].

## Conclusions

To our knowledge, this is the first reported case of long-term, disease-free survival following combination multi-visceral and metastatic resection of pancreatic adenocarcinoma treated with neoadjuvant and adjuvant FOLFIRINOX chemotherapy. This case highlights not only the potential benefit of surgery in the era of more effective chemotherapeutic regimes but also the dilemma faced by clinicians and MDT’s taking decisions on individual patients with isolated, hepatic metastases from pancreatic adenocarcinoma in the absence of randomized trials to support these treatment pathways. It is crucial that each case is evaluated on its own merits, all management decisions such as this are taken collectively by experienced clinicians from all disciplines, and that patients considered for these complex surgical interventions--often associated with high morbidity--are adequately counseled as to the potential risks in the context of the available evidence.
